# Comparison of three methods for ascertainment of contact information relevant to respiratory pathogen transmission in encounter networks

**DOI:** 10.1186/1471-2334-10-166

**Published:** 2010-06-10

**Authors:** James M McCaw, Kristian Forbes, Paula M Nathan, Philippa E Pattison, Garry L Robins, Terence M Nolan, Jodie McVernon

**Affiliations:** 1 Vaccine & Immunisation Research Group, Murdoch Childrens Research Institute & Melbourne School of Population Health, University of Melbourne, Parkville, VIC 3010 Australia

## Abstract

**Background:**

Mathematical models of infection that consider targeted interventions are exquisitely dependent on the assumed mixing patterns of the population. We report on a pilot study designed to assess three different methods (one retrospective, two prospective) for obtaining contact data relevant to the determination of these mixing patterns.

**Methods:**

65 adults were asked to record their social encounters in each location visited during 6 study days using a novel method whereby a change in physical location of the study participant triggered data entry. Using a cross-over design, all participants recorded encounters on 3 days in a paper diary and 3 days using an electronic recording device (PDA). Participants were randomised to first prospective recording method.

**Results:**

Both methods captured more contacts than a pre-study questionnaire, but ascertainment using the paper diary was superior to the PDA (mean difference: 4.52 (95% CI 0.28, 8.77). Paper diaries were found more acceptable to the participants compared with the PDA. Statistical analysis confirms that our results are broadly consistent with those reported from large-scale European based surveys. An association between household size (trend 0.14, 95% CI (0.06, 0.22), *P *< 0.001) and composition (presence of child 0.37, 95% CI (0.17, 0.56), *P *< 0.001) and the total number of reported contacts was observed, highlighting the importance of sampling study populations based on household characteristics as well as age. New contacts were still being recorded on the third study day, but compliance had declined, indicating that the optimal number of sample days represents a trade-off between completeness and quality of data for an individual.

**Conclusions:**

The study's location-based reporting design allows greater scope compared to other methods for examining differences in the characteristics of encounters over a range of environments. Improved parameterisation of dynamic transmission models gained from work of this type will aid in the development of more robust decision support tools to assist health policy makers and planners.

## Background

Mathematical models of infection are useful decision support tools for health policy makers choosing between alternative interventions to limit the spread of infectious diseases [1]. Increased focus on influenza pandemic preparedness and response in recent years has stimulated the development of agent-based models exploring alternative containment and mitigation strategies [2-4] that may be employed to limit disease transmission. Such models have highlighted uncertainty regarding critical transmission parameters describing mixing between age classes or social groups in heterogeneously mixing populations [3]. As model conclusions are often exquisitely sensitive to these parameter assignments [5,6], more recent models have drawn on European-derived data on age-based interactions to explore the impact of targeted immunisation strategies on Influenza A(H1N1) 2009 spread [7].

European researchers have developed paper diary tools to estimate the number and intimacy of conversational and physical encounters between individuals [8]. By these means, social mixing patterns within and between age groups have been characterised in a range of European countries [9]. The information obtained from paper diaries has been compared with retrospective web-based self-report [10] and while broadly consistent, prospective recording was more informative. Conversely, Glass et al compared the use of paper diaries with a classroom-completed survey and found the latter to be more consistently completed by school-aged individuals [11].

The timeliness and hence completeness of recording using paper diaries has been questioned in other applications. Stone and colleagues, using a paper diary electronically instrumented to capture opening and closure, were able to demonstrate a marked discrepancy between reported and actual compliance with completion of a pain diary [12]. Diaries were not even opened on 32% of study days; in contrast, 94% actual compliance was achieved with an electronic diary [12]. Similar comparison of paper and electronic tools in a range of health services research applications has consistently demonstrated improved completeness and accuracy when electronic devices are used [13]. Particular benefits are the ability to objectively assess timeliness of data entries [14], and ease and acceptability of use [15].

The primary aim of this study was to compare three different methods for the recording of social contacts likely to be sufficient for transmission of respiratory pathogens. Timeliness and completeness of entries in paper diary tools was compared to those entered in a hand-held electronic diary (PDA). We also assessed the ability of a questionnaire administered before study commencement to predict social encounters. Furthermore, we employed an explicitly location-based design across all three methods (pre-entry, paper, PDA) to aid in construction of bi-partite network models, in which interactions are characterised by the places in which they occur. Statistical characteristics of the recorded interactions were used for methodological assessment and comparison with European and other studies.

## Methods

### Recording of encounters

65 adult participants were asked to complete a pre-entry questionnaire detailing their anticipated activities and social contacts over three defined days (Wednesday, Friday, Sunday). They were then randomised to first use either a paper diary or personal digital assistant (PDA). Participants recorded their conversational or touch encounters over the three days before switching to the alternative method for the same days in the subsequent week. The study day commenced on waking and ended with sleep. The day of study commencement was not randomised, but varied with the day of enrolment between participants.

The diaries were designed to explicitly allow recording of social encounters within the context of locations visited by participants on a given study day (see section *Diary Tools *in the Additional file 1 for examples of the diary tools). Participants were asked to describe each new location (e.g. home, car, supermarket) and record the time of arrival and departure. The total number of people present and the number of people within arms length were also captured. While at a location, participants were asked to describe every substantive contact episode: those involving a two-way or small group conversational exchange of at least 3 words, or any skin-to-skin contact. Contacts were denoted by first name and initial, as well as occupation and usual place of residence, where known. In addition, the duration, location and intensity of the encounter (talk or physical contact) were recorded.

At the end of the study, participants were asked to rate relative difficulty and timeliness of paper and electronic diary completion.

This study was conducted between May and July 2008 and its conduct was approved by the University of Melbourne's Health Sciences Human Ethics Sub-Committee (ID 0721768.2). Written informed consent was obtained from all subjects prior to participation.

### Statistical analysis and sample size

The number and type (touch, talk) of contacts made was described across the whole dataset, for each category of location and for each participant. The data on both total recorded encounters and uniquely named contacts on a given day was modelled using a negative-binomial regression model (STATA 10, Stata Corporation, TX, USA 2007). The model was used to identify key participant (age, sex, household size, presence of child <18 years in the household) and methodological (diary type, day of the week, survey day, first method used) predictors of observed social encounters. Observations were grouped by participant using the 'cluster' command to ensure accurate reporting of variance, given repeated measures on the same individuals over time. Predictors, including interaction terms, found to be significant (P < 0.05) in univariate analyses (see section *Statistical Analyses *in the Additional file 1) were incorporated in the multivariate negative binomial regression model reported here.

Gross ascertainment differences between prospective diaries (paper and PDA) were assessed using the Bland-Altman test.

Assuming that the paper diary would capture a similar number of contacts to previous studies (mean 17, standard deviation 8) [8], the present protocol was powered, under a Gaussian model-assumption, to detect a difference of 3 contacts per day between recording methods, with 85% power and at 2-tailed alpha error level of 5%.

## Results

### Subject characteristics

**Figure 1:**
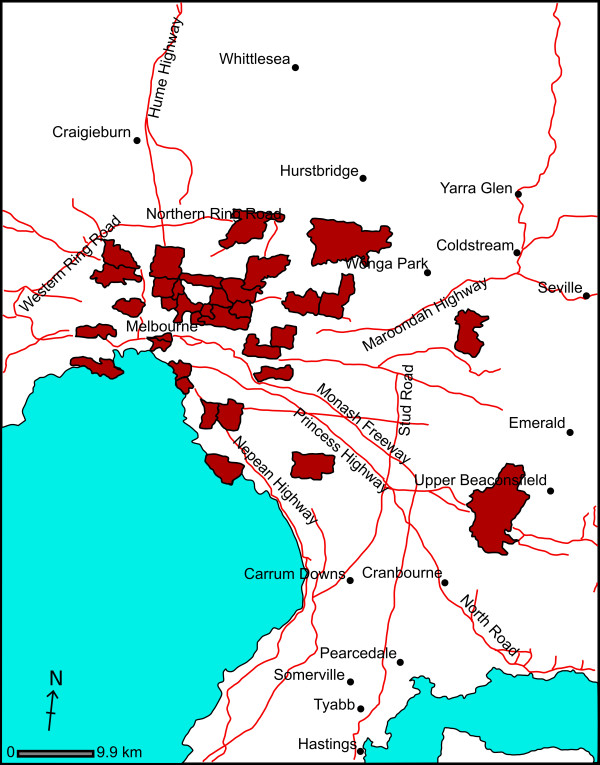
**Postcode of residence of study participants**. Postcode regions in the Melbourne metropolitan area are shaded to indicate the usual residence of study participants. Between 1 and 7 subjects lived in each of the highlighted regions. In addition, two participants came from regional Victoria (one each from Bendigo and Kyneton).

65 adult participants took part in the study. 54 were female. A large proportion were university employees engaged in health-related research. Others outside the academic sector became aware of the study by word of mouth. 13 of the subjects were aged 20-29 years, 14 were 30-39 years, 22 were 40-49 years, 11 were 50-59 years and 5 were older than 60 years. The participants lived in households containing between 1 and 6 individuals (median of 3), of which half contained at least one member less than 18 years of age. As can be seen from Figure 1, respondents resided in a diverse range of postcode districts within the metropolitan area of Melbourne, a city of nearly 4 million people in the state of Victoria, Australia. A further 2 participants were recruited from regional Victoria.

### Contact recordings

**Figure 2:**
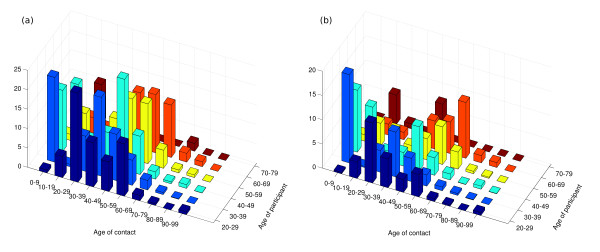
**Mixing within and between age groups**. The average number of encounters recorded in the paper diary between subjects and contacts in defined age categories is shown for a) all encounters and b) encounters involving physical contact. It should be noted that while averages are reported, the number of observations within each age group upon which these are based varies between 1 (70-79) and 22 (40-49). As can be seen, most contacts occur between individuals of the same age. Further, adults aged between 30-39 years mix with children under 10, presumably in the household. Similarly, 40-49 year olds have a large number of contacts with children and teenagers. Where only encounters involving some physical contact are considered (b), mixing between parents and children becomes more prominent.

Almost 13,000 contact episodes were recorded across all three methods: 5,544 in the paper diary, 4,392 in the PDA and 3,035 in the pre-entry questionnaire. Approximately half of all encounters recorded by any means involved some form of physical contact. 55% of all reported contacts were made with members of the same sex. As seen in previous studies [9], mixing was nominally assortative (within age-group), although no formal analysis was performed due to small study numbers and the methodological focus of this study. In addition, large numbers of contacts were made between adults aged 30-50 years and children, with more of these involving touch than within-age group encounters (Figure 2).

**Figure 3:**
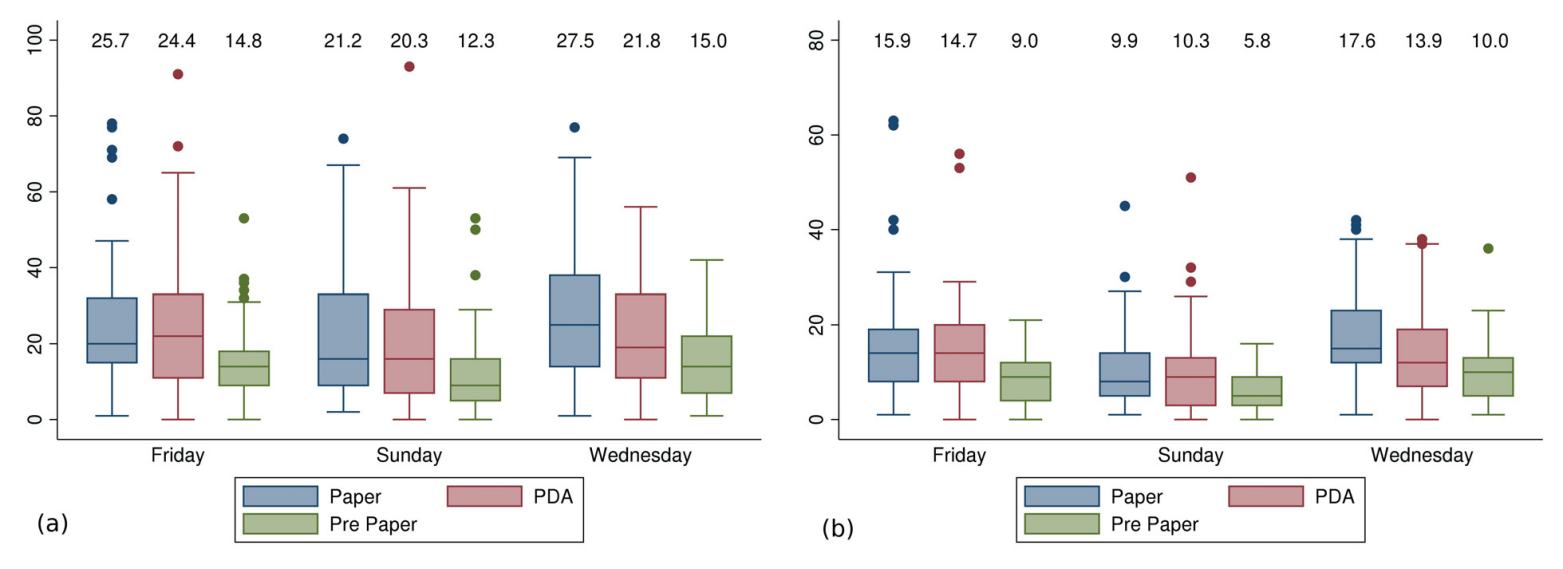
**Number of individual contacts per day, by source and day of the week**. For each subject, the number of recorded contacts per day was determined. For each day of the week studied, we show the median number of contacts recorded in the paper diary, PDA diary and entry questionnaire (Pre Paper). Upper and lower bounds of the boxes reflect the interquartile range, whiskers define upper and lower adjacent values. We also report the mean value above each box plot. We report total contacts (a) and the number of unique names (b).

As repeated encounters with the same nominated individual were common, similar comparisons were made using the number of uniquely identified contacts. Table 1 and Figure 3 summarise the findings by method and day of week.

**Table 1:** Number of encounters/nominated individuals for each participant by recording source and day (mean; median and interquartile range; range)

	All encounters			Nominated individuals		
	**Paper**	**PDA**	**Pre-Paper**	**Paper**	**PDA**	**Pre-Paper**

**Friday**	25.7 20 (15,32) (1-78)	24.4 22 (11,33) (0-91)	14.8 14 (9,18) (0-53)	15.9 14 (8,19) (1-63)	14.7 14 (8,20) (0-56)	9.0 9 (4,12) (0-21)

**Sunday**	21.2 16 (9,33) (2-74)	20.3 16 (9,33) (2-74)	12.3 9 (5,16) (0-53)	9.9 8 (5,14) (1-45)	10.3 9 (3,13) (0-51)	5.8 5 (3,9) (0-16)

**Wednesday**	27.5 25 (14,38) (1-77)	21.8 19 (11,33) (0-56)	15.0 14 (7,22) (1-42)	17.6 15 (12,23) (1-42)	13.9 12 (7,19) (0-38)	10.0 10 (5,13) (1-36)

### Summary of the multivariate negative-binomial model

Results from the negative-binomial model are shown in Tables 2 and 3, applied to all encounters (Table 2) and individually nominated contacts (Table 3). The model fit is shown in section *Statistical Analyses *in the Additional file 1. For comparison across methods, we take encounters recorded in the paper diary as our reference, i.e. Table 2 indicates that use of either the PDA (coefficient = -0.12, *P *= 0.034) or pre-entry questionnaire (coefficient = -0.57, *P *< 0.001) compared to the paper diary was predictive of fewer recordings. Wednesday was used as the reference for the effect of day, and study day 1 (for a given method) as the reference for the effect of study day.

**Table 2:** Multivariable negative binomial regression model for all recorded encounters

Variable	Coefficient	Standard Error	P value
PDA (ref paper)	-0.12	0.057	0.034

Pre-entry questionnaire (ref paper)	-0.57	0.064	<0.001

Friday (ref Wed)	0.03	0.059	0.609

Sunday (ref Wed)	-0.17	0.077	0.026

Survey day 2 (ref 1)	-0.09	0.064	0.141

Survey day 3 (ref 1)	-0.23	0.072	0.001

Household size (trend)	0.14	0.041	<0.001

Presence of child in the household	0.36	0.100	<0.001

Standard error accounts for clustering by subject. Negative binomial overdispersion parameter (mean, 95% CIs): α = 0.39 (0.31, 0.49).

**Table 3:** Multivariable negative binomial regression model for individually nominated contacts

Variable	Coefficient	Standard Error	P value
PDA (ref paper)	-0.11	0.057	0.057

Pre-entry questionnaire (ref paper)	-0.55	0.055	<0.001

Friday (ref Wed)	-0.06	0.062	0.318

Sunday (ref Wed)	-0.44	0.084	<0.001

Survey day 2 (ref 1)	-0.07	0.064	0.246

Survey day 3 (ref 1)	-0.23	0.073	0.001

Household size (trend)	0.13	0.037	<0.001

Presence of child in the household	0.16	0.086	0.064

Standard error accounts for clustering by subject. Negative binomial overdispersion parameter (mean, 95% CIs): α = 0.31 (0.24, 0.39).

### Comparison of reporting sources

The number of encounters made by each participant on a given day was compared across the 3 recording methods. Table 1 and Figure 3 demonstrate that subjects consistently underestimated their anticipated contacts in the pre-entry questionnaire (visual inspection). More contacts were recorded in the paper diary than the PDA on Wednesdays (*P *= 0.004, Wilcoxon matched-pairs signed-rank test for difference). These differences between reporting sources were significant in a multivariate negative-binomial regression model (Tables 2 and 3, Variables 'PDA' and 'Pre-entry questionnaire' have statistically significant negative coefficients) that examined the contribution of a range of survey and participant characteristics to the number of recorded encounters.

34 participants were randomised to use the paper diary first, with the remaining 31 commencing recordings with the PDA before switching to the alternative method. An additional term was included in the multivariate model to examine the impact of this sequence of recording method on reporting, and test for a participant learning or 'burn-out' effect. No significant effect was observed.

### Comparison of weekdays surveyed

Table 1 and Figure 3 demonstrate a greater number of contacts made on weekdays as opposed to weekend days. Wednesday and Friday appeared the same. The observed difference in encounters between Wednesday and Sunday was significant in the multivariate model (Tables 2 and 3, Variable 'Friday' is not significant while 'Sunday' has a statistically significant negative coefficient).

We were further interested to see whether there was an effect of the first, second or third study day on recording, again indicative of either a learning effect or 'burnout'. It should be noted that first study day was not randomised and 33 subjects commenced recordings on a Wednesday, 16 on a Friday and 16 on a Sunday. As a consequence, day of the week was strongly correlated with survey day (Pearson's correlation coefficient 0.26, *P *< 0.001). There was, however, a significant reduction in recording by the third study day in a model that also took day of the week into account (Tables 2 and 3, Variable 'Survey day 2' is not significant while 'Survey day 3' has a statistically significant negative coefficient. Also see section *Summary of contact data by survey day *in the Additional file 1), suggesting that compliance declined over time.

### Contribution of other demographic characteristics

Participant age and sex were not found to be significant in the univariate analysis. However, household size and presence or absence of a child (<18 years) were found to be significant explanatory variables. No evidence for an interaction between household size and presence of a child was found, despite household size being significantly associated with presence of a child (*P *< 0.001).

These associations were borne out by study of characteristics of outliers in the sample. The four participants who recorded the most contacts by either means (of whom two overlapped - one a nurse, the other a student) all resided in households whose size was at or above the 75^th ^percentile and contained children. Respondents recording the least contacts were less likely to live with children, and two were retired. Ages of high and low respondents ranged across the full distribution seen among participants.

### Non-saturation of contacts over recording days

**Figure 4:**
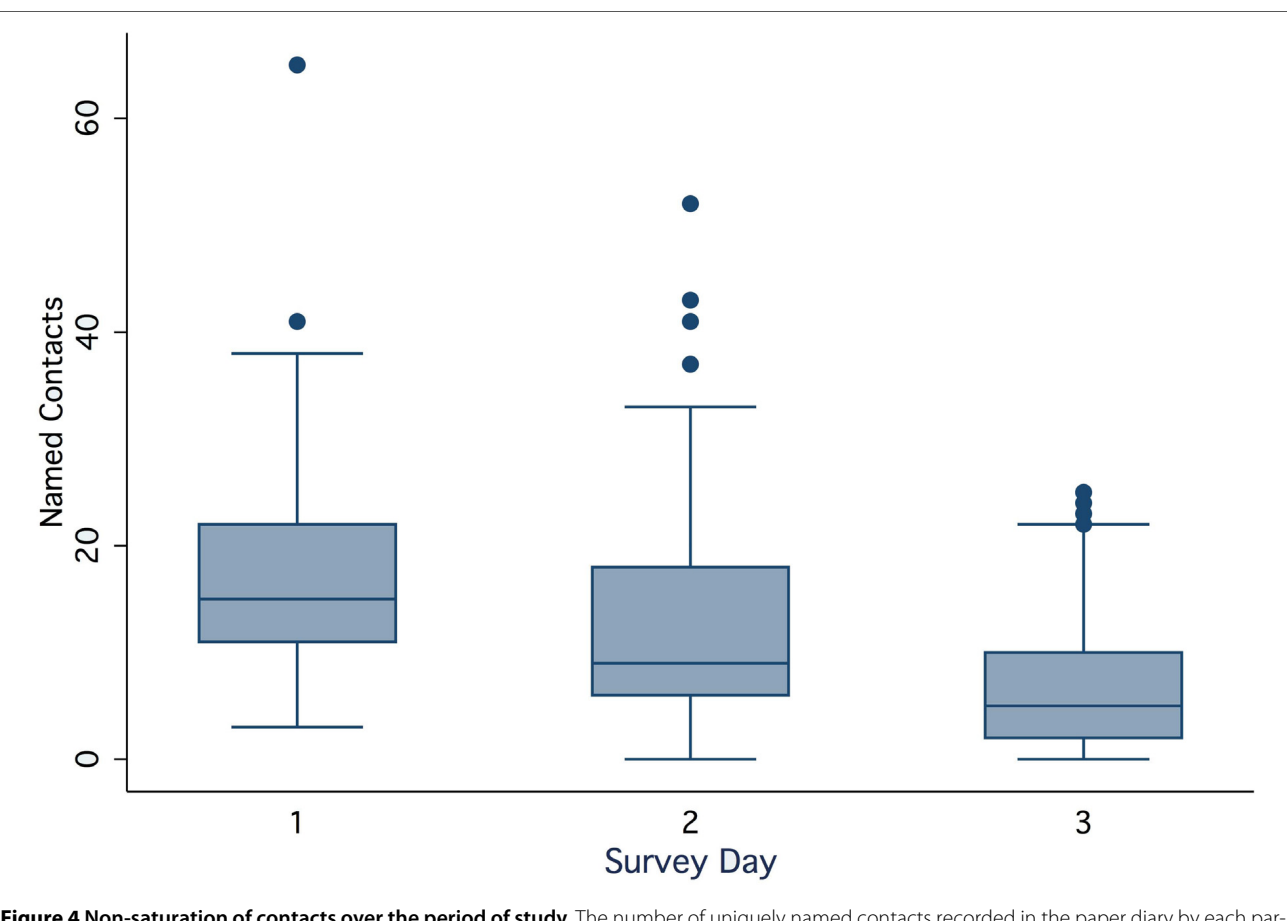
**Non-saturation of contacts over the period of study**. The number of uniquely named contacts recorded in the paper diary by each participant (median total of 33) was calculated for each of the three study days. As can be observed, new contacts were still being made by the third study day by the majority of participants.

The number of new individuals encountered over successive study days was examined using the paper diary record, as this was the most complete. Participants recorded a median of 33 different nominated contacts (range 6-115) over the 3 days. As can be observed in Figure 4, new contacts were still made by the majority of participants on the third study day. Given the observed differences between encounters by day of the week, we compared numbers of newly named individuals by survey day (1, 2 or 3) on each of Sunday, Wednesday and Friday and observed similar trends (see section *Non-saturation of contacts over recording days *in the Additional file 1).

### Recordings by location type

**Figure 5:**
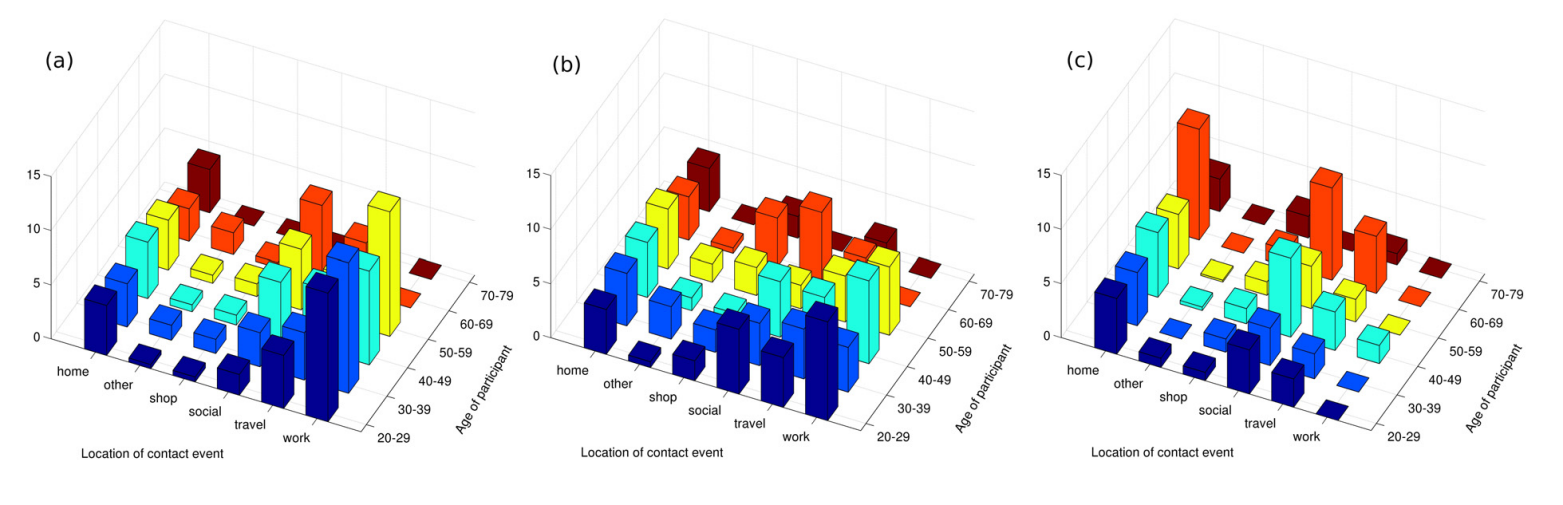
**Location in which encounters took place**. The average number of encounters per participant recorded in the paper diary within each age category was described for each of the following locations: 'work', 'travel', 'social', 'shop', 'home' and 'other' on Wednesday (a), Friday (b) and Sunday (c). It should be noted that while averages are reported, the number of observations within each age group upon which these figures are based varies between 1 (70-79) and 22 (40-49). As can be seen from comparison of locations reported on Wednesday (a) and Sunday (c), a predictable shift from predominance of work-based mixing to home-based and social encounters is observed over the weekend. On Wednesdays (a), however, levels of physical contact in the home environment are almost equivalent to those reported in the workplace (data not shown). Wednesday (a) and Friday (b) are more similar.

Further to the difference in number of named contacts between Wednesday and Sunday, a change in the location of contacts made on these days was also observed (Figure 5). Again, Wednesday and Friday showed similar location profiles. As can be seen from comparison of locations reported on Wednesday and Sunday, a shift from predominance of work-based mixing to home-based and social interaction was observed over the weekend. When contacts involving physical contact of any kind were compared, however, numbers of encounters in the workplace were almost equivalent to those reported in the home environment on Wednesdays (data not shown).

### Evaluation of recording methods

**Figure 6:**
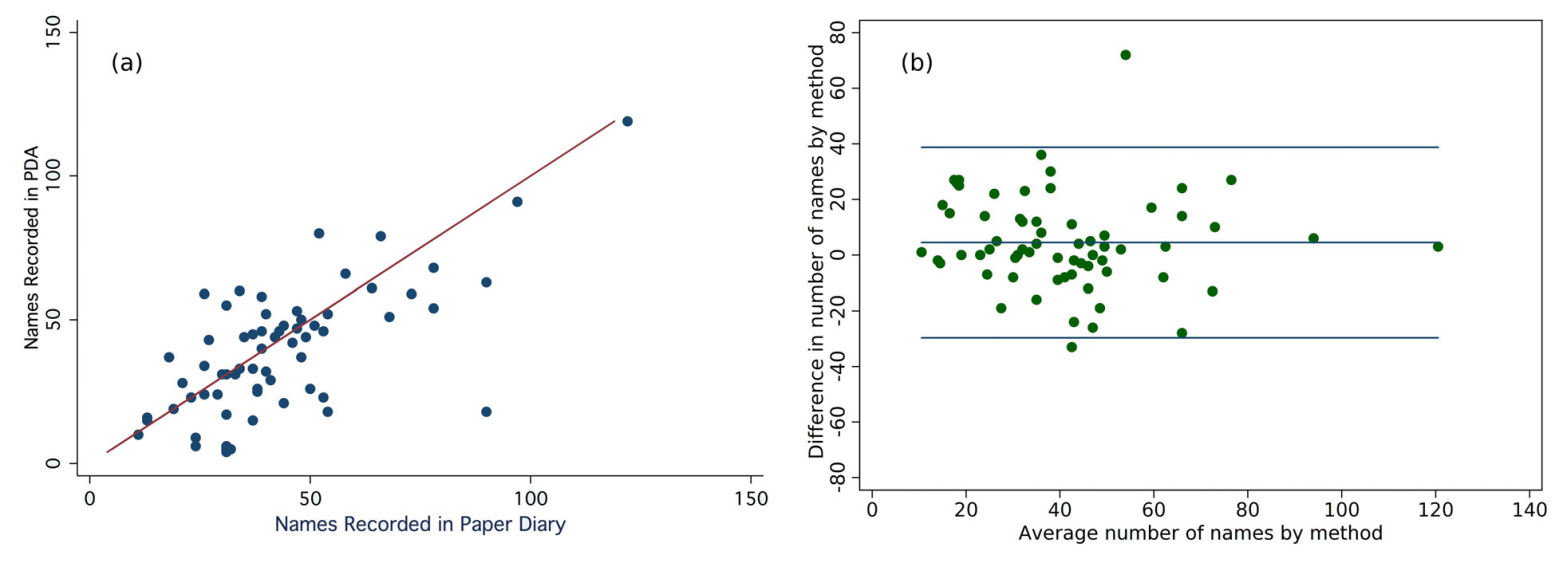
**Correlation between Paper and PDA entries for each participant**. Despite variable self report of the difficulty and timeliness of data entry using either paper or PDA, close correlation (0.68) was found between participants' recording of encounters by either means, indicating that both methods are relatively successful in capturing participants encounters (a). The Bland-Altman plot shows a systematic and significant underreporting using the electronic diary compared to paper (b). The horizontal rules show the mean difference (4.52 (0.28, 8.77), and the reference range for difference (-29.7 to 38.8).

Participants reported no difficulty in remembering to take either the paper or electronic diary with them each day. Almost all reported their estimates of encounters as 'about right' by either means. There was, however, a distinction in self-reported ease of use with the majority of respondents (63%) describing the paper diary as 'easy' to use, with contrasting divided opinions on the PDA (30% 'difficult' and 35% 'easy'). Despite this perceived difficulty, good within-subject concordance was found between the two prospective reporting sources (correlation coefficient 0.69) indicating that both tools are adequate (Figure 6a). Consistent with findings reported in Tables 2 and 3, the mean difference, as assessed by the Bland Altman method [16], in the number of names recorded between paper and PDA methods was 4.52 (95% CI 0.28, 8.77) (Figure 6b).

More participants reported filling out the diary 'within 1 hour' when using the paper form (32%) rather than the PDA (16%). Most entries were made 'several hours later' regardless of the type of diary (35% paper, 37% PDA), with more participants delaying data entry to the end of the day with the PDA (23%) compared with the paper record (13%). This self-report was concordant with the actual time from encounter to data entry as automatically captured by the electronic diary, with a mean delay of 3.6 hours (median <1 hour). Again, the timeliness of data entry did not significantly affect the number of named individuals recorded by either reporting source (univariate analysis, data not shown).

## Discussion

This study used an explicitly location-dependent ascertainment method to capture social encounters. We were convinced of the validity of participants' self-reported encounters relevant to respiratory pathogen transmission by the rich narrative thread describing the day's activities captured within the diaries. More encounters were captured using the paper diary than other methods, with participants consistently reporting ease of use and timely data entry by this means. Moreover, respondents uniformly underestimated social encounters when asked to predict their contacts over the survey period (pre-entry questionnaire), justifying the need for contemporaneous diary recording.

The study's crossover design reduced between-individual variation when comparing the number of recorded contacts across prospective (paper, PDA) methods. Potential biases due to training, learning or 'burn-out' effects were minimised by ensuring that all participants were recruited and enrolled by a single research assistant (PMN) and by randomising subjects to commence recording using either the paper or electronic diary. Future protocols will additionally include randomisation of the first recording day [17], to minimise interactions between day of the week and survey day.

Several factors may have contributed to the relatively poor acceptance of the electronic diary in this study compared with others, despite the relatively high representation of health-based researchers in this convenience sample who might be anticipated to be familiar with similar recording tools. Firstly, our location-based methodology may place a greater burden on participants than other study protocols. Secondly, the custom-built software designed for this study could potentially have been made more accessible with participant input over a longer development phase, although the close correlation observed between paper and PDA recordings for each subject suggested that most participants persisted with the electronic diary in spite of perceived difficulty. With the increasing use of 'smartphone' technologies, development of high-quality PDA-style software presents as an emergent challenge to field-based researchers. In relation to the study's objectives, the fact that respondents reported longer delays in entering contacts into the PDA went against the rationale for its use in other settings [13], as improved timeliness of recording is desirable to minimise recall bias.

Definitive validation of recorded contacts by some form of external non-participant based observation would be desirable, but aside from privacy concerns, would conceivably change behaviour. As with other studies to date designed to measure the social interactions relevant to respiratory disease transmission, this study was not able to capture illness or exposure events. Mikolajczyk et al [18] investigated whether contact counts for a group of school children in Germany were predictive of infection in the past 6 months, with no effect of household size or contacts observed. It remains a key challenge to design and deploy study protocols able to capture both an individual's dynamic social-network and their concurrent disease status (e.g. susceptible, exposed, infectious). Such data would provide definitive validation of the methods employed both here and in other studies.

The diary method developed for this protocol differed from tools used in other studies as location was the focal point for defining and describing each new set of recorded encounters. In consequence, and in contrast to earlier work [8], repeated encounters were commonly described. For the purpose of assessing the comparability of our findings with other studies, we have identified the number of uniquely named individuals encountered on each day. The utility of signalling a change of location as a stimulus to recording is suggested by the relatively high number of contacts recorded by our study participants, compared with respondents from the European Union [9]. For example, the mean (and standard deviation) number of reported contacts per day was 11.74 (7.67) in the United Kingdom and 19.77 (12.27) in Italy. The dynamic nature of contacts over the three surveyed days, with new casual contacts still being made on the third day of diary recording, concords with earlier findings from the United Kingdom [17].

In an analysis of the POLYMOD data, Kretzschmar et al [19] have classified individuals into seven distinct contact profiles, based on the locations in which they predominantly mix. The analysis will allow for the characterisation of how an infection spreads between locations, fulfilling a similar aim to our locations-based data for parameterising bi-partite networks of social contacts and locations. The diary format used here, whereby the 'intensity' of contact events in each separate location is captured through multiple measures (all people in the location, those at arms length, those with a recorded contact), will allow comparative analysis of different measures of intensity when constructing similar contact profiles.

The usefulness of recording the intensity of contact is confirmed by consistent observation of closer mixing in home and social settings than work environments [17]. However, the importance of sampling to observed within and between-age mixing patterns is demonstrated by contrasting findings of university-based studies [8,17,20] with those containing a larger proportion of family households, such as ours. While the convenience sample employed in the present protocol was not representative of metropolitan Melbourne, our findings were more concordant with results from a large-scale European study, in demonstrating a high level of child-adult mixing within families [9]. In addition, we too observed an association between household size and the total number of reported contacts [9]. These findings highlight the importance of deriving population samples that are representative not only of age, but also the household size distribution within a given country and setting.

With others, we observed clear differences between weekend and weekdays, both in the number and location of encounters. Due to budgetary and resource limitations, we restricted collection to three study days, choosing to have only one weekend day (Sunday). The absolute number of contacts observed was similar to findings from the European POLYMOD surveys [9]. Clearly it is desirable to capture information about individuals over as many days as possible in order to assess true daily variation. However we have confirmed the observation of reduced compliance with recording over time [21], which undermines the validity of repeated observations. Hens et al also observed decreased compliance over just two days using a modification of the POLYMOD survey in Belgium [22]. Defining the optimal sampling time frame is a necessary trade off and may be driven by the primary study question of interest. For example, it may be desirable to estimate the total number of potential contacts made by an individual infected with a given respiratory pathogen, in which case the duration of the infectious period may determine the number of study days, with appropriate caveats regarding data quality.

Our results confirm the importance of household-level mixing to providing opportunities for the spread of infection both within and between age groups. Further data collection is required to supplement this pilot study in order to aid parameterisation of models describing heterogeneity of population mixing in the Australian urban context. How these results compare to similar European studies will be of interest.

Models of infectious disease spread are of use to policy makers aiming to predict the likely impact of interventions to reduce disease transmission targeted at specific age groups or social settings. Discussion of the possible benefit of school closure to mitigate the spread of pandemic influenza is a timely example and reflects current uncertainty regarding the contribution of spread in different settings to outbreak dynamics [23,24].

We have reported both mean and median encounters in Figure 3 as our data serves dual purposes. Mean encounters are the most appropriate input into stratified compartmental mathematical models of disease-transmission, which assume homogenous contact numbers within age-strata. Furthermore, in such models it is the structure of the Who-Acquires-Infection-From-Whom (WAIFW) matrix, used to capture the relative propensity of mixing between strata, that is of key importance [25]. The main concern in this context is with the distribution of contacts, rather than their absolute number and so comparison of data recording sources should therefore focus on a comparison of the inferred WAIFW matrices. In contrast, stochastic agent based simulations or network-based models of disease transmission directly model the distribution of cases. Furthermore, in this context the absolute number of contacts is important, for example in modelling the degree distribution of network nodes. As such, characterisation of our data by median and interquartile ranges of the observations is appropriate.

Variation in behaviour is likely to explain, at least in part, differences in the number of secondary transmission events between individuals. Published stochastic models [26] have examined the consequence of allowing for variation in the offspring distribution, while such effects can be routinely incorporated into agent-based and network-based models. Furthermore, our location-based methodology, in contrast to other methods [9] captures the different environments in which repeat encounters are likely to occur. Spatial agent-based simulations [2,4] model the movement of agents (individuals) from location to location over time, currently parameterised using census data. Our study method provides data of exactly this type, with associated proxy measures of exposure-risk given by the intensity of contact events within each location. Similarly, our location-based data is key to the development of pathogen transmission models based on bi-partite graphs of social-networks by location. Of course, the relative importance of different locations to pathogen transmission is a complex function of time, number of contacts, 'intensity' of contacts and the pathogen itself, with no definitive studies as yet to reject or accept one model structure over another.

## Conclusions

We have reported on the use of three different diary tools for the recording of social encounters relevant to respiratory pathogen transmission. Our results indicate that prospective diary tools provide more complete data than a pre-entry questionnaire. The study's location-based reporting design allows greater scope compared to other methods for examining differences in the characteristics of encounters over a range of environments.

Now that we are confident of the quality of our data collection tool, further analysis of the detailed time-use information gathered will be undertaken, with a focus on how different measures of 'contact-intensity' may influence the characterisation of key locations for contact mixing. Improved parameterisation of dynamic transmission models gained from work of this type will aid in the development of more robust decision support tools to assist health policy makers and planners.

## Abbreviations

PDA: personal digital assistant;

## Competing interests

The authors declare that they have no competing interests.

## Authors' contributions

JMcC, JMcV and KF performed the statistical analyses and interpreted the results. PN coordinated the study, recruiting all subjects and collating the data. JMcC, JMcV, PP, GR and TN conceived the study and designed the diary collection tools. JMcV and JMcC wrote the initial drafts of the manuscript. All authors contributed to and approved the final manuscript.

## Author's information

Vaccine & Immunisation Research Group, Murdoch Childrens Research Institute & Melbourne School of Population Health, The University of Melbourne, Victoria, Australia (James M McCaw, Paula M Nathan, Kristian Forbes, Terence M Nolan, Jodie McVernon)

School of Behavioural Science, The University of Melbourne, Victoria, Australia (Philippa E Pattison, Garry L Robins)

## Pre-publication history

The pre-publication history for this paper can be accessed here:

http://www.biomedcentral.com/1471-2334/10/166/prepub

## Supplementary Material

Additional file 1 **Supplementary Material**. Supplementary Material for Comparison of three methods for ascertainment of contact information relevant to respiratory pathogen transmission in encounter networks Click here for file
